# Reply to Adrian, G. Comment on “Russo et al. Does Tumor Volume Have a Prognostic Role in Oropharyngeal Squamous Cell Carcinoma? A Systematic Review and Meta-Analysis. *Cancers* 2022, *14*, 2465”

**DOI:** 10.3390/cancers14174302

**Published:** 2022-09-02

**Authors:** Elena Russo, Andrea Costantino, Giuseppe Mercante, Giuseppe Spriano, Armando De Virgilio

**Affiliations:** 1 Department of Biomedical Sciences, Humanitas University, Via Rita Levi Montalcini 4, 20090 Pieve Emanuele, Italy; 2 Otorhinolaryngology Unit, IRCCS Humanitas Research Hospital, Via Manzoni 56, 20089 Rozzano, Italy

The authors raised some interesting points [1] related to our previously published meta-analysis [2]. In this study, we analyzed the prognostic impact of the tumor volume in oropharyngeal squamous cell carcinomas (OPSCC), distinguishing between the primary (pTV) and nodal (nTV) tumor volumes. We found that both parameters were not associated with worse overall survival (OS), while the nTV was associated with worse tumor control, as demonstrated by the analysis of the disease-free survival (DFS) and locoregional control (LRC).

The authors stated that the pooled estimates were obtained by combining the continuous and dichotomized hazard ratios (HRs), and they suggested that we may have found different results by considering the two parameters separately. As suggested, we performed a meta-analysis differentiating between the studies reporting continuous HRs and those reporting dichotomized HRs. In particular, we either extracted the HRs and 95% confidence intervals (CIs) directly from each study, if reported, or estimated them indirectly using the method described by Tierney et al. [3]. The HRs were log-transformed before pooling effect size estimates were measured. Then, cumulative HRs with the 95% CI were calculated by the inverse variance method using a random-effects model. 

As shown in Table 1, the results obtained by the stratified analyses are not significantly different from those shown in our published study. In particular, high nTV was found to be associated with a statistically significant poorer DFS and LRC in the HR_continuous_ group. On the other hand, the other analyses identified no significant impacts of pTV on tumor control or survival. Moreover, high nTV was not associated with worse survival. 

The authors stated that some individual studies showed a significant impact of the tumor volume, analyzed using dichotomized or continuous variables. Adrian et al. [4] identified worse tumor control in patients with a high tumor volume only in a subgroup of patients who underwent conventional fractionation radiotherapy, but the difference was no more significant upon merging all the data. Accordingly, Lok et al. [5] identified a worse survival rate with a high tumor volume, but the results were not statistically significant after pooling the data of 669 patients from 4 studies. As correctly stated by the authors, the weight assigned in the meta-analysis is dependent on the standard error (SE). In particular, a smaller SE corresponds to a smaller sampling error and the consequent better estimation of the true overall effect. Moreover, the SE is inversely proportional to the increase in the sample size, which is then taken into account in the assignment of the weight of each study. Specifically, the variance of each effect size is obtained using the square of the SE, and the inverse of the variance is used to determine the weight of each study, since a lower variance indicates the higher precision of the estimate. In the random-effects model, the inverse variance method is used to calculate an adjusted random-effects weight for each observation in order to obtain a pooled conservative estimate [6,7]. The distinction between the HRs reported as either continuous or dichotomized allow us to obtain more homogeneous SEs between studies, but the pooled effect size did not significantly differ from the results of our previous analysis.

We thank the authors for their valuable comments on our study, which allowed us to improve the precision of our analysis. The current literature data do not suggest that the tumor volume represents a prognostic factor of high clinical significance in regard to the OPSCC. However, the low quality of the available studies and the high level of inter-study heterogeneity requires further attention in order to better define the roles of pTV and nTV.

## Figures and Tables

**Table 1:** Pooled outcomes.

	pTV					nTV				
	N.studies	N.pts	HR	95% CI	*p* Value	N.studies	N.pts	HR	95% CI	*p* Value
**OS continuous**	1	91	1.02	0.96–1.08	0.19	1	91	1.01	0.89–1.15	0.48
**OS dichotomized**	4	669	1.70	0.72–4.01	0.14	1	340	0.99	0.59–1.67	0.97
**DFS continuous**	3	175	1.01	0.99–1.03	0.15	3	175	1.02	1.01–1.03	<0.01 *
**DFS dichotomized**	3	288	1.40	0.46–4.27	0.32	NA	NA	NA	NA	NA
**LRC continuous**	2	122	1.05	0.53–2.10	0.51	2	122	1.02	1.01–1.03	<0.01 *
**LRC dichotomized**	3	614	1.82	1.00–3.32	0.05	NA	NA	NA	NA	NA

* Statistically significant results. Abbreviations: pTV, primary tumor volume; nTV, nodal tumor volume; OS, overall survival; DFS, disease-free survival; LRC, locoregional control; HR, hazard ratio; CI, confidence interval.

## Data Availability

The data presented in this study are available on request from the corresponding author.
